# Mesenchymal Stromal Cell-Derived Extracellular Vesicles Attenuate Dendritic Cell Maturation and Function

**DOI:** 10.3389/fimmu.2018.02538

**Published:** 2018-11-09

**Authors:** Monica Reis, Emily Mavin, Lindsay Nicholson, Kile Green, Anne M. Dickinson, Xiao-nong Wang

**Affiliations:** Haematological Sciences, Institute of Cellular Medicine, Newcastle University, Newcastle upon Tyne, United Kingdom

**Keywords:** extracellular vesicles, mesenchymal stromal cells, immunomodulation, dendritic cells, immunomodulation, microRNA

## Abstract

Mesenchymal stromal cells (MSCs) are potent regulators of immune responses largely through paracrine signaling. MSC secreted extracellular vesicles (MSC-EVs) are increasingly recognized as the key paracrine factors responsible for the biological and therapeutic function of MSCs. We report the first comprehensive study demonstrating the immunomodulatory effect of MSC-EVs on dendritic cell (DC) maturation and function. MSC-EVs were isolated from MSC conditioned media using differential ultracentrifugation. Human monocyte-derived DCs were generated in the absence or presence of MSC-EVs (20 ug/ml) then subjected to phenotypic and functional analysis *in vitro*. MSC-EV treatment impaired antigen uptake by immature DCs and halted DC maturation resulting in reduced expression of the maturation and activation markers CD83, CD38, and CD80, decreased secretion of pro-inflammatory cytokines IL-6 and IL-12p70 and increased production of anti-inflammatory cytokine TGF-β. MSC-EV treated DCs also demonstrated a diminished CCR 7 expression after LPS stimulation, coupled with a significantly reduced ability to migrate toward the CCR7-ligand CCL21, although they were still able to stimulate allogeneic T cell proliferation *in vitro*. Through microRNA profiling we have identified 49 microRNAs, which were significantly enriched in MSC-EVs compared to their parent MSCs. MicroRNAs with known effect on DC maturation and functions, including miR-21-5p, miR-142-3p, miR-223-3p, and miR-126-3p, were detected within the top 10 most enriched miRNAs in MSC-EVs, with MiR-21-5p as the third highest expressed miRNA in MSC-EVs. *In silico* analysis revealed that miR-21-5p targets the *CCR7* gene for degradation. To verify these observations, DCs were transfected with miR-21-5p mimics and analyzed for their ability to migrate toward the CCR7-ligand CCL21 *in vitro*. MiR-21-5p mimic transfected DCs showed a clear trend of reduced CCR7 expression and a significantly decreased migratory ability toward the CCL21. Our findings suggest that MSC-EVs are able to recapitulate MSC mediated DC modulation and MSC-EV enclosed microRNAs may represent a novel mechanism through which MSCs modulate DC functions. As MSCs are currently used in clinical trials to treat numerous diseases associated with immune dysregulation, such as graft-versus-host disease and inflammatory bowel disease, our data provide novel evidence to inform potential future application of MSC-EVs as a cell-free therapeutic agent.

## Introduction

Mesenchymal stromal cells (MSCs) are currently used in clinical trials to treat a range of immune dysregulation associated diseases (1–3). The ability to modulate a wide-range of immune responses is a well-recognized property of MSCs, through mechanisms largely involving secretion of paracrine factors (4). Increasing evidence has emerged recently suggesting that MSC-derived extracellular vesicles (MSC-EVs) are the principle biological factors through which MSCs exert their key functions in immunomodulation and tissue regeneration. MSC-EVs, including exosomes and microvesicles, are a heterogeneous population of bilipid membrane nanoparticles encapsulating a plethora of bioactive factors, including, proteins (cytokines, membrane receptors, growth factors and enzymes) and genetic materials (mRNAs and microRNAs) (5). They function as key intercellular signaling mediators to elicit biological responses via horizontal transfer of their bioactive cargo into recipient cells (5, 6).

Recent studies have shown MSC-EV mediated immunomodulation by targeting T cells, B cells, and NK cells (7–13). *In vitro* and *in vivo* studies have also shown that MSC-EVs induce an anti-inflammatory phenotype in macrophages, characterized by the production of anti-inflammatory cytokines IL-10 and consequent generation of regulatory T cells (8, 14). However, despite the pivotal role that dendritic cells (DCs) play in initiating and regulating immune responses (15) and the fact that DCs are a key target for MSC mediated immunomodulation, no comprehensive study has been reported so far to demonstrate the modulatory effect that MSC-EVs may have on the maturation and function of DCs. Furthermore, little is known about the mechanisms of action by which MSC-EVs exert their immunomodulatory effect. Increasing attention has been given to MSC-EV enclosed microRNAs for their roles in post-transcriptional regulation of gene expression through mRNA silencing. MSC-EV enclosed microRNAs have been shown to play important roles in the protection of tissue damage and promotion of tissue repair in animal models of myocardial ischemia, acute kidney injury, and osteoarthritis (6, 16–20). To date the potential contribution of MSC-EV enclosed microRNAs in immunomodulation of DC function remains unknown.

In this study, we investigated whether MSC-EVs are capable of recapitulating the previously well-established immunomodulatory effects that MSCs have on DC maturation and function (21, 22) by examining the phenotypic and functional features of MSC-EV treated DCs in comparison to their untreated counterparts, including the expression of maturation/activation markers, the ability to uptake antigen and stimulate allogeneic T cells, as well as the profile of cytokines secreted by DCs and T cells stimulated with treated and untreated DCs. MSC-EV treated DCs were further examined for their ability to migrate via the CCR7 dependent pathway. We also profiled the microRNAs encapsulated in MSC-EVs and performed *in silico* and *in vitro* analysis to elucidate the mechanism of action of MSC-EV mediated immunomodulation.

## Materials and methods

### MSC culture and characterization

Human bone marrow-derived MSCs were generated using standard plastic adherence method from healthy donor bone marrow aspirates (surplus to hematopoietic stem cell transplantation, obtained from the Newcastle Cellular Therapy Facility, Newcastle upon Tyne, UK). In brief, bone marrow mononuclear cells (MNCs) were isolated by density gradient centrifugation using Lymphoprep™ (Axis-Shield). MNCs were then plated at a density of 2 × 10^7^ cells/flask in T-25 tissue culture flasks in basal medium containing Dulbecco's modified eagle medium, 100 IU/ml penicillin, 100 μg/ml streptomycin, 2 IU/ml heparin and 2 mM L-glutamine (all from Sigma-Aldrich), supplemented with 5% human platelet lysate (hPL; PLTMax, Mill Creek Lifesciences) (23). The cells were cultured for 3 days at 37°C in a 5% CO_2_ incubator. The non-adherent cell fraction was discarded, and fresh medium was added to the adherent cells. Medium was refreshed every 3 days and cells were passaged when the culture reached 70–80% confluence. MSCs at passage 3 were characterized according to the criteria described by the International Society of Cellular Therapy (ISCT) (24) and used in all experiments throughout this study.

### MSC-EV isolation

MSC-EVs were collected from MSC conditioned medium by differential ultracentrifugation, as previously described (25). EV-depleted medium was prepared by overnight ultracentrifugation at 100,000 × g of basal medium supplemented with 10% hPL. When passage 3 MSCs reached 90% confluence, cells were washed twice with phosphate buffered saline (PBS, Sigma-Aldrich) and cultured in EV-depleted medium, at a final concentration of 5% EV-depleted hPL, for a further 48 h prior to MSC-EV isolation. The conditioned medium was then centrifuged at 400 × g for 5 min at 4°C to exclude detached cells and debris. The resulting supernatant was centrifuged at 2,000 × g for 20 min at 4°C, transferred to ultracentrifuge tubes (Beckman Coulter) and centrifuged sequentially at 10,000 × g for 45 min and at 100,000 × g for 90 min at 4°C using a 45Ti rotor (Beckman Coulter) in a BECKMAN L8-80 ultracentrifuge (Beckman Coulter). The MSC-EV pellet was washed in 60 ml of PBS then re-suspended in at least 100 μl of sterile PBS and stored at −80°C.

### MSC-EV characterization

Collected MSC-EVs were characterized based on their morphology, particle size and surface protein expression. EV morphology was visualized using transmission electron microscopy (TEM). Briefly, 5 μl of PBS suspended MSC-EVs were adsorbed for 30 s onto a carbon-coated, glow discharged grid. Excess liquid was removed with a filter paper (Whatmann no. 50, Sigma-Aldrich). Samples were stained with 1% uranyl acetate for 30 s. Excess uranyl acetate solution was removed and the MSC-EV loaded carbon-coated grids were dried under a lamp. Grids were examined using a Philips CM100 Compustage (FEI) transmission electron microscope and digital images were collected using an AMTCCD camera (Deben) housed in Electron Microscopy Research Services, Newcastle University. The particle size and concentration were measured by nanoparticle tracking analysis (NTA). Briefly, MSC-EV pellets were diluted at 1:100–1:500 ratios with sterile-filtered PBS and analyzed using a Nanosight LM10 (Malvern), as described by the manufacturer's protocol. Three 1-min measurements were taken for each sample. The acquired data was processed using NTA 2.3 software (Malvern). EV surface markers CD63, CD9 and CD81 were analyzed by flow cytometry. The volume of EVs suspension equivalent to 5 μg of protein [measured with the microBCA protein assay kit (ThermoFisher Scientific)], were coated onto 4-μm aldehyde/sulfate latex beads (LifeTechnologies) by overnight incubation on a rotary wheel at RT. The reaction was stopped by incubation with 1 M glycine (Sigma-Aldrich) for 30 min at RT. The MSC-EV-bead complex was washed twice with PBS with 0.5% fetal calf serum (FCS, Invitrogen), and incubated with CD63 PE (H5C6), CD9 PerCPCy5.5 (M-L13) and CD81-APC (JS-81) antibodies or corresponding isotype controls (all from BD Biosciences) for 20 min at 4°C. Following further washes in PBS with 0.5% fetal calf serum data was acquired using a BD FACS Canto II cytometer and analyzed with FlowJo software (Tree Star).

### Magnetic isolation of CD3^+^ T cells and CD14^+^ monocytes

Peripheral blood mononuclear cells (PBMCs) were obtained from healthy donors by gradient centrifugation over Lymphoprep™. CD3^+^ T cells and CD14^+^ monocytes were immuno-magnetically isolated by depletion of non-T cells using the Pan T cell isolation kit (Miltenyi Biotec) and positive selection using human CD14 microbeads (Miltenyi Biotec), respectively. Highly purified (>90% purity) CD3^+^ T cells and CD14^+^ monocytes were used in the subsequent experiments.

### Generation of monocyte-derived DCs and co-culture with MSC-EVs

Mature and immature monocyte-derived dendritic cells (mDC and iDC, respectively) were generated from magnetically isolated CD14^+^ monocytes (26). The cells were cultured in complete medium (RPMI 1640 with 10% FCS, 2 mM L-glutamine and 100 IU/ml penicillin, 100 μg/ml streptomycin) supplemented with IL-4 and GM-CSF (both at 50 ng/ml; Immunotools) for 7 days. MSC-EVs at a concentration of 20 μg/ml were added to the DC generation culture on day 3, when fresh medium containing the same concentrations of IL-4 and GM-CSF was replenished. Mature DCs were generated by adding LPS (100 ng/ml; Sigma) to the DC culture on day 6. The EV-treated immature and mature DCs (iDC-EV and mDC-EV) were harvested on day 7 and used in the subsequent experiments.

### T cell proliferation

The influence of MSC-EVs on T cell proliferative response was evaluated in two experimental settings. Initially MSC-EVs were added directly into the co-cultures of CFSE labeled CD3^+^ T cells and allogeneic mDCs, with the co-cultures without added MSC-EVs as controls. Subsequently, MSC-EVs were added into the DC generation cultures as described above. Mature DCs, with or without previous exposure to MSC-EVs, were co-cultured with CFSE labeled allogeneic CD3^+^ T cells. The same T cell: DC ratio (10:1) was used in both experimental settings. The amount of MSC-EVs used in both settings was identical (20 μg/ml), which was pre-determined by dose titration (Supplementary Figure S1). This MSE-EV dose was used in all functional analysis throughout this study. The levels of T cell proliferation were determined by CFSE dilution using flow cytometry on day 5 of the co-culture, with the frequency of CFSE^low^ cells as readout for the levels of T cell proliferation.

### MSC-EV uptake by immune cells

MSC-EVs were labeled with PKH26 Red Fluorescence Cell Linker Kit (Sigma-Aldrich), following the manufacturer's protocol with minor modifications. Briefly, 20 μg/ml of MSC-EVs were pelleted at 100,000 × g for 90 min and re-suspended in 1 ml of Diluent C to which 2 μl of PKH26 was added. Diluent C containing PKH26 without MSC-EVs was used as a negative control. After a 20-min incubation at RT in the dark, the reaction was stopped with 1 ml of 1% bovine serum albumin (BSA; Sigma-Aldrich). Labeled MSC-EVs were washed twice with filtered PBS at 100,000 × g for 90 min then re-suspended in complete medium and added to the co-culture of allogeneic CD3^+^ T cells and mDCs. After 24 h of incubation at 37°C in a 5% CO_2_ humidified atmosphere, the cells were washed in PBS, suspended in serum-free RPMI 1640 and seeded onto a poly-L-lysine coated coverslip (2 × 10^5^ cells/coverslip). Cells were incubated for 15 min at 37°C to allow attachment to the coverslip and fixed with 2% paraformaldehyde for 15 min at RT. Cells were washed 2 × with PBS and blocked with antibody diluent (PBS with 0.1% Triton X (Sigma-Aldrich) and 0.5% BSA), for 30 min at 4°C. The cells were then incubated overnight with mouse anti-human CD3 antibody (1:100, BD Biosciences) at 4°C. Coverslips were washed 2 × with PBS containing 0.1% Triton and 0.1% BSA and incubated with the secondary antibody (goat anti-mouse Dy649, 1:300; Jackson Immunoresearch) for 2 h at 4°C. The coverslips were washed 2 × and incubated with HLA-DR FITC (1:20, BD Biosciences) for an additional 4 h, washed 2 × and mounted with Vectashield mounting medium containing DAPI (Vector Laboratories). The samples were imaged using Axioimager Z2 fluorescence microscope with axiovision software V4.8 (Zeiss).

### Flow cytometry analysis

The staining for cell surface proteins were performed following standard protocols by incubation of cells with pre-optimized concentrations of antibodies at 4°C for 20 min in FACS buffer (PBS with 2% FCS and 1 mM EDTA). Flow cytometry data were acquired on BD FACS Canto II cytometer and analyzed with FlowJo software version X (Tree Star). Unless otherwise stated all antibodies were supplied by BD Biosciences: CD25 APC (M-A251) or CD25 PECy7 (2A3); CD8 APC (SK1); CD4 V500 (RPA-T4), CD3 PerCPCy5.5 (SK7), CD38 PE (HIT2); CD80 PE (L307.4); CD83 FITC (HB15e); CD86 PECy7 (FUN-1), HLA-DR PerCP (L243); CCR7 PECy7 (G043H7; Biolegends). Appropriate isotypes were used as controls.

### Phagocytosis assay

The ability of immature DC to uptake antigens was assessed by incubation of immature DCs (5 × 10^4^) with FITC-dextran (1 mg/ml; Sigma-Aldrich) in basal media for 1 h at 37°C including a control incubated at 4°C to exclude extracellular binding of FITC-dextran. Cells were then washed three times in cold FACS buffer and analyzed by flow cytometry.

### Cytokine quantification

The level of cytokines was quantified in the culture supernatants collected from either DC generation cultures on day 7 or DC and T cell co-cultures on day 5 using cytometric bead array flex kit, according to the manufacturer's instructions (BD Biosciences). DC supernatants were assessed for IL-6, IL-10, IL-12p70, and TGF-ß1 whilst DC-T cell co-culture supernatants for IL-6, IFNγ, TNFα and IL-2. Data was acquired on a FACS Canto II and analyzed with FCAP software (BD Biosciences).

### DC migration

DC migration was assessed as described by Anderson et al. (27), with minor modifications (27). Briefly, migration was measured using a transwell system (pore size 8 μm, Corning Lifesciences), mDCs (1 × 10^5^) were added in the upper chamber, and medium, with or without CCL21 (250 ng/ml, R&D systems) in the lower chamber. After 24 h-incubation at 37°C migrated mDCs in the lower chamber were harvested and counted using a Neubauer chamber (Celeromics). Migration efficiency was calculated from the percentage of the input cells that had migrated. The percentage of mDCs which had migrated toward the medium without CCL21 served as a negative control. The migration conditions used in this study were pre-optimized (Supplementary Figure S2).

### MSC-EV miRNA profiling

Total RNA in MSC-EVs was purified using the Total Exosome RNA and Protein isolation kit (Thermo Fisher Scientific). Total RNA from the parent MSCs was purified from cell lysates using the Qiagen RNAeasy mini kit, as per the manufacturer's instructions. The concentration and quality of total RNA from MSC and MSC-EVs was determined using Bioanalyzer 2100 with the RNA 6000 Nano and Pico kits, respectively (Agilent technologies). At least 2 ng of total RNA were used as input for the nCounter® Human miRNA Expression assay V3 kit (NanoString Technologies). The miRNA expression profiles were then analyzed using the nSolver software V2.5 (NanoString Technologies) according to the manufacturer's instructions. Samples were normalized to the geometric mean of the 100 miRNAs with the highest counts and a microRNA was considered as differentially expressed when its expression exhibited a fold increase >1.5 and a *p*-value < 0.05 as determined by the nSolver software using Student's *t*-tests.

### Quantitative real time PCR

Validation of MSC-EV and MSC miRNA expression profiles was performed by qRT-PCR. Total RNA extraction and quality was assessed as aforementioned. The miRNA specific copy DNA synthesis was carried out using the SensiFAST™ probe HI-ROX master mix (Bioline) and the Taqman® primer probe sets for: hsa-mir-21-5p (*5*′*-UAGCUUAUCAGACUGAUGUUGA-3*′), hsa-miR-223 (*5*′*-UGUCAGUUUGUCAAAUACCCCA-3*′), has-miR142-3p (*5*′*-UGUAGUGUUUCCUACUUUAUGGA-3*′), hsa-miR-126 (*5*′*-UCGUACCGUGAGUAAUAAUGCG-3*′) and U6 snRNA control (*5*′*-GTGCTCGCTTCGGCAGCACATATACTAAAATTGGAACGATACAGAGAAGATTAGCATGGCCCCTGCGCAAGGATGACACGCAAATTCGTGAAGCGTTCCATATTTT-3*′), (all from Thermo Fisher Scientific).

### DC transfection

The miRIDIAN microRNA hsa-miR-21 mimic and the miRIDIAN microRNA transfection control with Dy547 were purchased from Dharmacon. The siRNA dried pellets were re-suspended in RNase free water to a stock solution of 20 μM, then stored at −80°C. The transfection of DCs was performed based on a lipid-siRNA transfection method as previously described (28). Briefly, 3 × 10^5^ CD14^+^ monocytes were seeded in 24-well plates in complete media supplemented with 50 ng/ml of GM-CSF and IL-4. On day 3 of the culture, the lipid-siRNA complexes were formed by combining 117.5 μls of warm serum-free RPMI with 3.75 μls of HiPerFect transfectant (Qiagen) and 3.75 μls of siRNA stock solutions (final concentration of 200 nM). The lipid-siRNA mixture was incubated for 15–20 min at RT with constant mixing to enhance the formation of the complexes. 125 μls of the lipid-complex were added to one side of a well of a tilted 24-well plate and day 3 DCs were added directly into the lipid-siRNA complexes and the plate was gently swirled to ensure uniform distribution of the lipid-siRNA complexes then incubated at 37°C and 5% CO_2_ for 4 h. The transfection was then stopped with RPMI supplemented with GM-CSF and IL-4 and cells were kept in culture until day 7 as aforementioned. Transfection efficiency and viability of the cells was monitored on days 4, 5, 6, and 7 of DC generation and the effect of miR-21 overexpression on DC maturation and function was assessed.

### Statistical analysis

Unless otherwise stated, all statistical analysis was carried out using GraphPad Prism version 6 (GraphPad Software). Mann-Whitney U tests or Student's *t*-tests were used to determine statistical significance. Differences were considered statistically significant at a *p*-value < 0.05.

## Results

### MSC and MSC-EV characteristics

**Figure 1:**
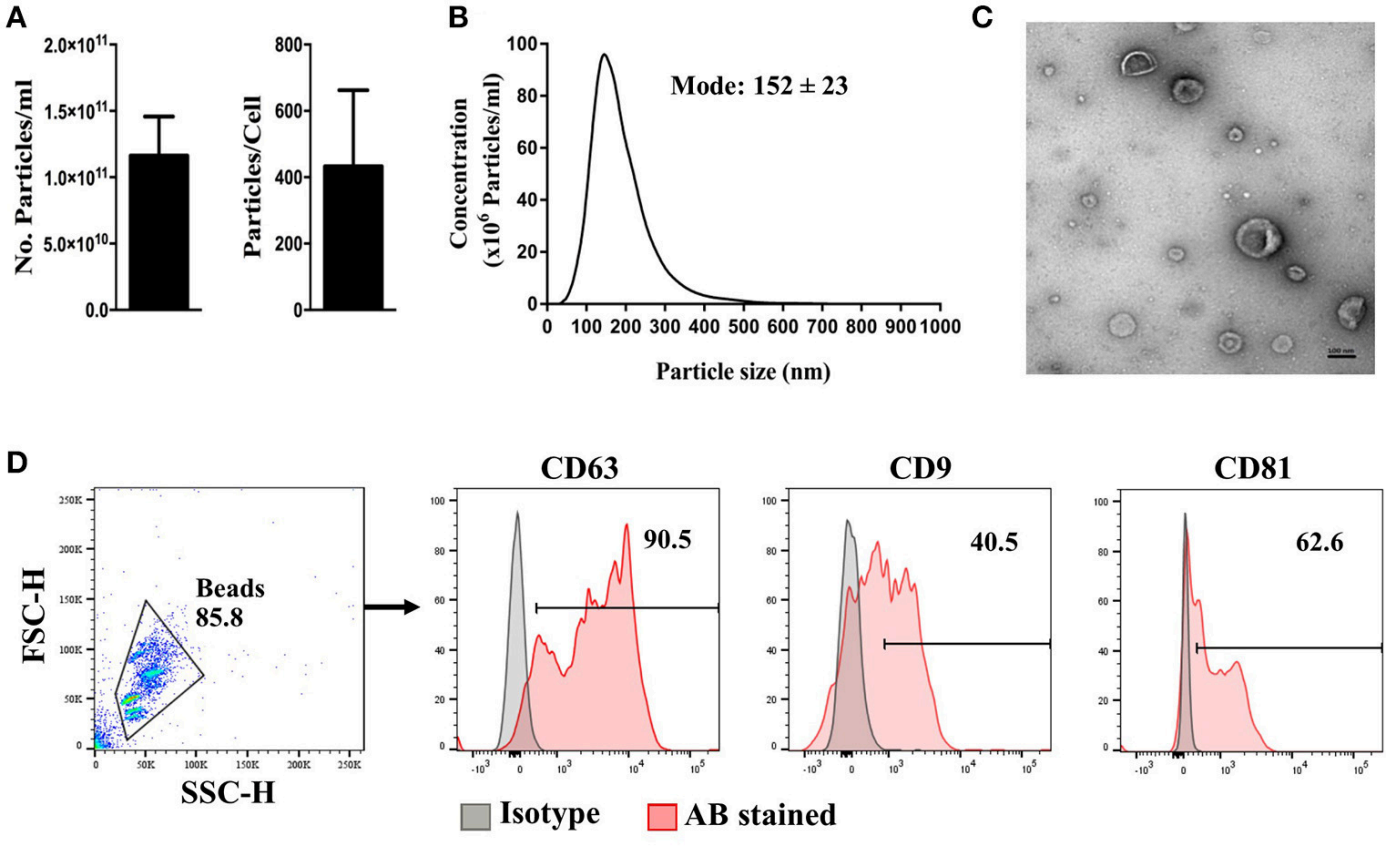
Characteristics of MSC-derived EVs. MSC-EVs were isolated from MSC conditioned medium by differential ultracentrifugation. Isolated EVs were assessed for **(A)** particle concentration, **(B)** particle size distribution, and **(C)** morphology using nanoparticle-tracking analysis and transmission electron microscopy, respectively. Flow cytometry was used to comfirm the expression of EV signature markers **(D)**. The scale bar represents 100 nm. The error bars represent mean ± SEM of four independent experiments.

MSCs were generated from healthy bone marrow aspirates and expanded in xeno-free conditions. The generated MSCs were confirmed as being compliant with the ISCT criteria (24) prior to be used for EV purification (Supplementary Figure S3). MSC-EV purification yielded an average of 1.16 × 10^11^ ± 2.95 × 10^10^ particles/ml, or 432 ± 229 particles/cell (Figure 1A). Purified MSC-EVs showed a modal size of 152 ± 23 nm (Figure 1B), and exhibited the expected cup-shaped morphology, as assessed by TEM (Figure 1C). MSC-EVs positively expressed common transmembrane proteins enriched in EVs, including CD63, CD9, and CD81 (Figure 1D).

### MSC-EVs preferentially target DCs

**Figure 2:**
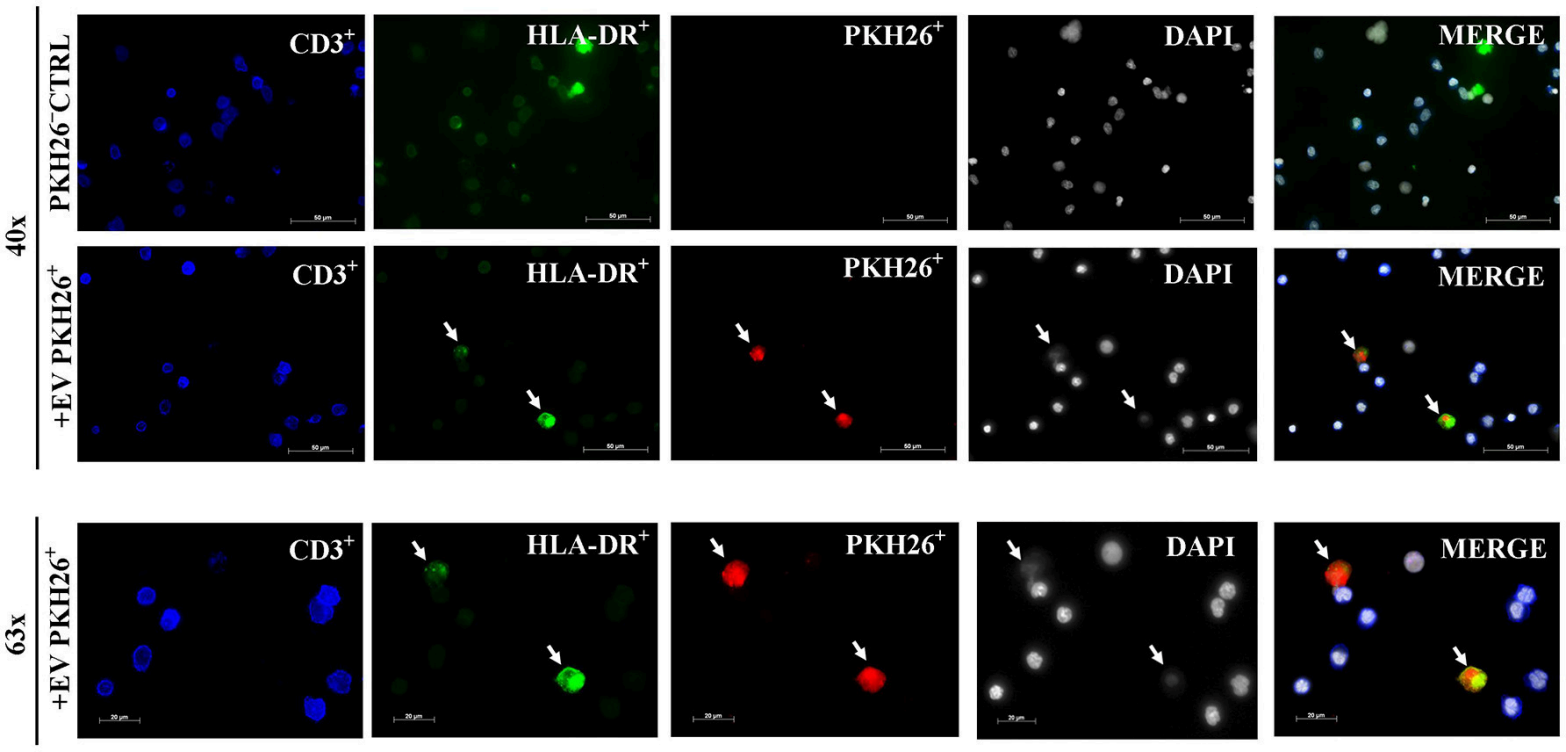
MSC-EVs preferentially target DCs. Representative images from confocal microscopy showing that EV-PKH26^+^ labeled MSC-EVs (red) are closely associated with DCs (green). No uptake of MSC-EVs by CD3^+^ T cells (blue) was detected. The scale bars represent 50 and 20 μm.

To identify whether MSC-EVs primarily target DCs or T cells, PKH26 labeled MSC-EVs were added into the co-culture of purified T cells and mDCs then visualized the spatial association of MSC-EVs with DCs and T cells. We found a predominant association between MSC-EVs and DCs detected by the fluorescence staining overlap of PKH26 labeled MSC-EVs (red) and HLA-DR labeled DCs (green). No association was detected between the labeled MSC-EVs and CD3^+^ T cells (Figure 2).

### MSC-EVs impair antigen uptake by immature DCs

**Figure 3:**
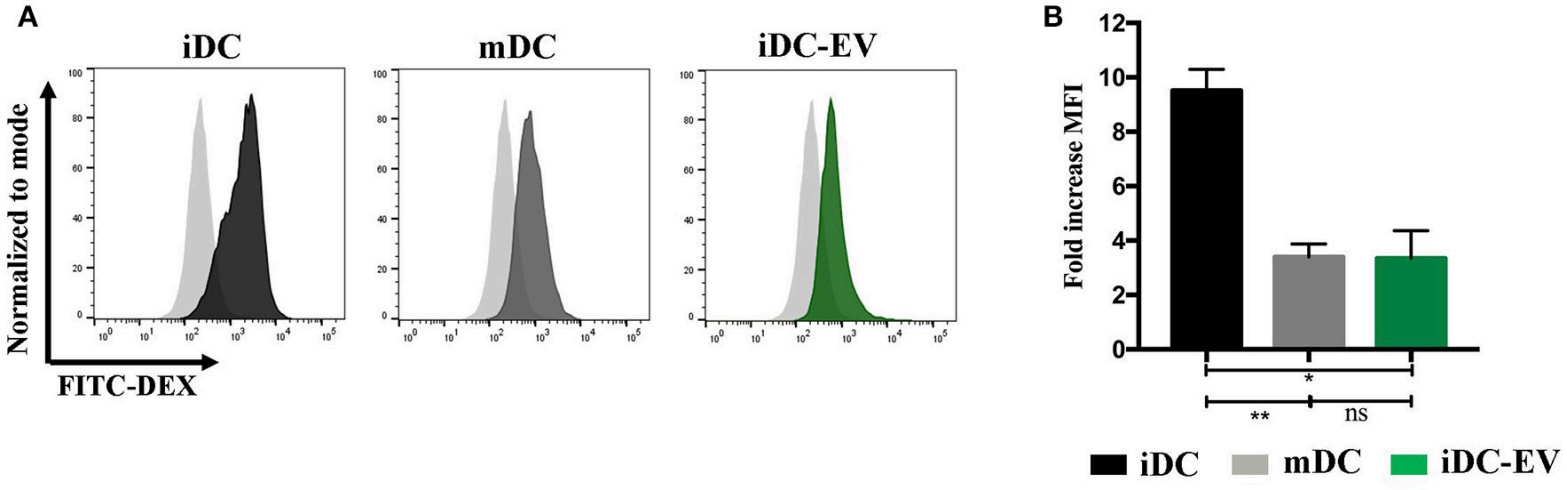
MSC-EVs impair antigen uptake by immature DCs. FITC dextran uptake was used to assess antigen uptake by immature, mature and MSC-EV treated immature DCs. **(A)** Representative histograms and **(B)** cumulative data of FITC dextran uptake as assessed by flow cytometry. Pale gray histograms represent isotype control. Results express mean ± SEM of four independent experiments (*p < 0.005, **p < 0.01).

Immature DCs (iDCs) effectively capture and process antigens, which can be assessed by their uptake of FITC-dextran. Upon DC maturation following the stimulation with LPS (a TLR4 ligand), a function switch occurs from antigen capture and processing to predominantly presenting antigens to T cells (29). As expected, iDCs showed significantly higher levels of FITC dextran uptake than the mDCs (*p* = 0.003). MSC-EV treated iDCs (iDC-EV) showed a decreased ability to take up FITC dextran (*p* = 0.012; Figure 3), suggesting that MSC-EVs can exert a modulatory effect on DC function by blocking the first step of DC maturation.

### MSC-EVs alter DC phenotype and function

**Figure 4:**
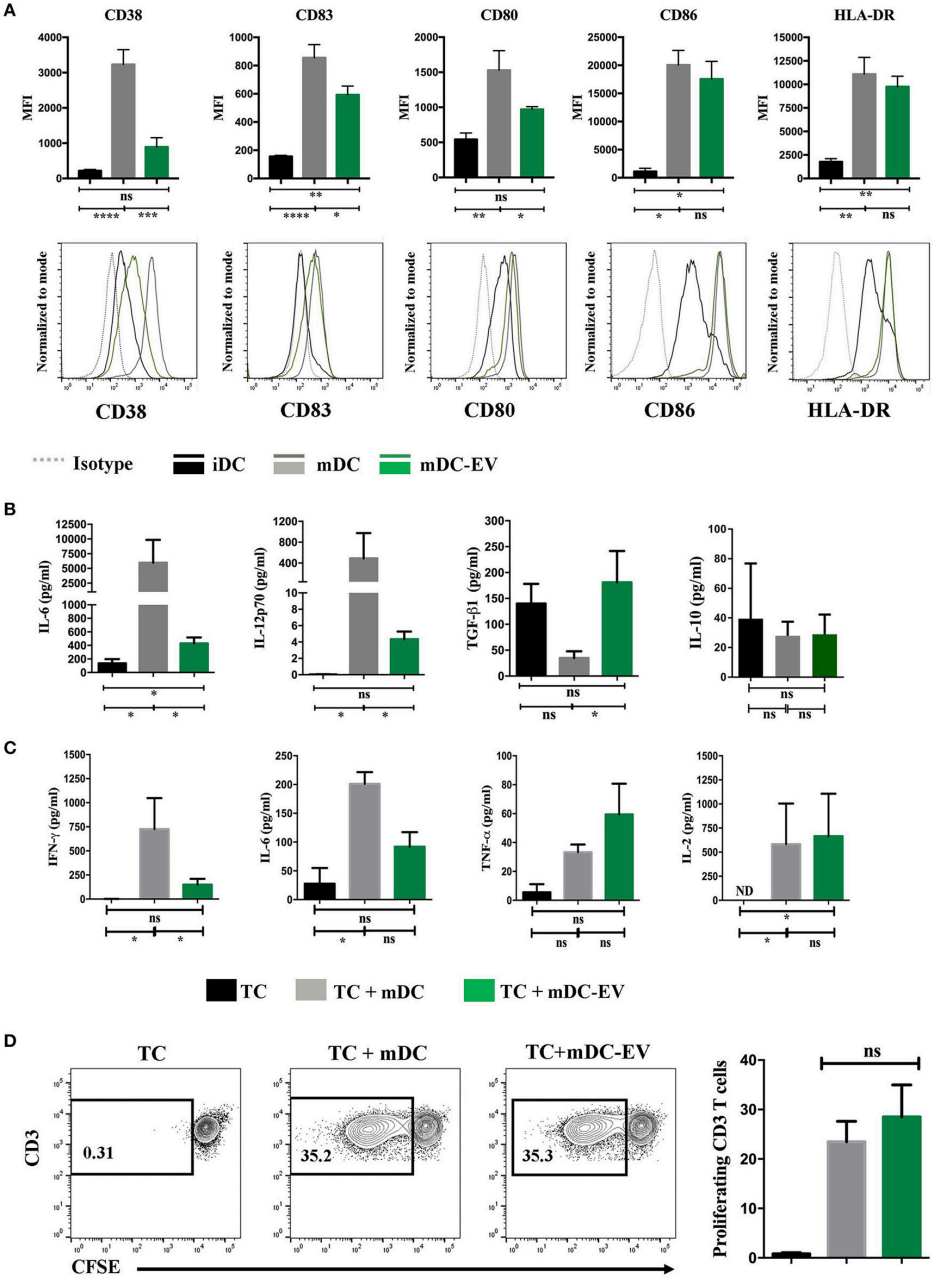
MSC-EVs modulate DC maturation and cytokine secretion. **(A)** Cumulative (top) and representative (bottom) data showing expression of surface markers CD38, CD83, CD80, CD86 and HLA-DR as determined by flow cytometry on immature, mature and MSC-EV treated mature DCs. **(B)** Cell culture supernatants collected from iDC, mDC and mDC-EVs were analyzed for the levels of IL-6, IL-12p70, TGF-β and IL-10. **(C)** Supernatants collected from the co-culture of CD3^+^ T cells with either mDCs or mDCs-EVs were analyzed for the levels of IFN-γ, IL-6, TNF-α and IL-2. T cells cultured with medium alone served as a control. **(D)** Representative (left) and cumulative (right) data showing allogeneic CD3^+^ T cell proliferation stimulated with mDCs and mDC-EVs as assessed by flow cytometry analysis of CFSE dilution. Data represent mean ± SEM of 4–6 independent experiments. *p < 0.05, **p < 0.01, ***p < 0.001, ****p < 0.0001. ND, not detectable.

We next examined the effect of MSC-EV treatment on DC maturation and activation by assessing their expression of CD83, CD38, co-stimulatory molecules CD80 and CD86, as well as HLA-DR. Mature and immature DCs showed expected high or low levels of all the aforementioned surface markers, respectively (*p* < 0.05; Figure 4A). MSC-EV treated mDCs expressed a markedly reduced expression of CD83, CD38 and co-stimulatory molecules CD80 compared to the untreated mDCs (*p* = 0.009, 0.05, and 0.03, respectively). No significant difference was detected in the expression of CD86 and HLA-DR between MSC-EV treated and untreated mDCs (Figure 4A).

Upon LPS stimulation mature DCs secrete high levels of pro-inflammatory cytokines, IL-6 and IL-12 (30). To verify the impact that MSC-EVs may have on mature DC cytokine secretion following LPS stimulation the levels of IL-6, IL-12-p70, IL-10, and TGF-β were measured in the culture supernatants of iDCs, mDCs, and MSC-EV treated mDCs. The levels of IL-6 production by MSC-EV treated mDCs was markedly lower than that secreted by untreated mDCs (*p* = 0.01) but higher than that secreted by iDCs (*p* = 0.03). MSC-EV treated mDCs produced the IL-12-p70 at a level that was over 100-fold lower than that produced by untreated mDCs (*p* = 0.036) but no significant difference to that produced by iDCs (*p* > 0.05). Importantly, MSC-EV treated mDCs showed an increased production of the anti-inflammatory cytokine TGF-β compared to untreated mDCs (*p* = 0.05). No significant difference was observed in IL-10 production across all DC groups (Figure 4B). To evaluate the functional effect of MSC-EV treated DCs on T cell responses we stimulated allogeneic T cells with MSC-EV treated DCs and measured T cell cytokine secretion and T cell proliferation. The results showed that T cells stimulated with MSC-EV treated DCs secreted lower levels of IL-6 and IFN-γ compared to those co-cultured with untreated DCs (*p* = 0.05 for both) while no significant difference was observed in TNFα and IL-2 production between the two groups (*p* > 0.05 for both; Figure 4C). However, MSC-EV treated DCs did not show a suppressive effect on allogeneic T cell proliferation compared to the T cells stimulated with untreated mDCs (Figure 4D).

### MSC-EVs impair DC migration via suppression of CCR7 expression

**Figure 5:**
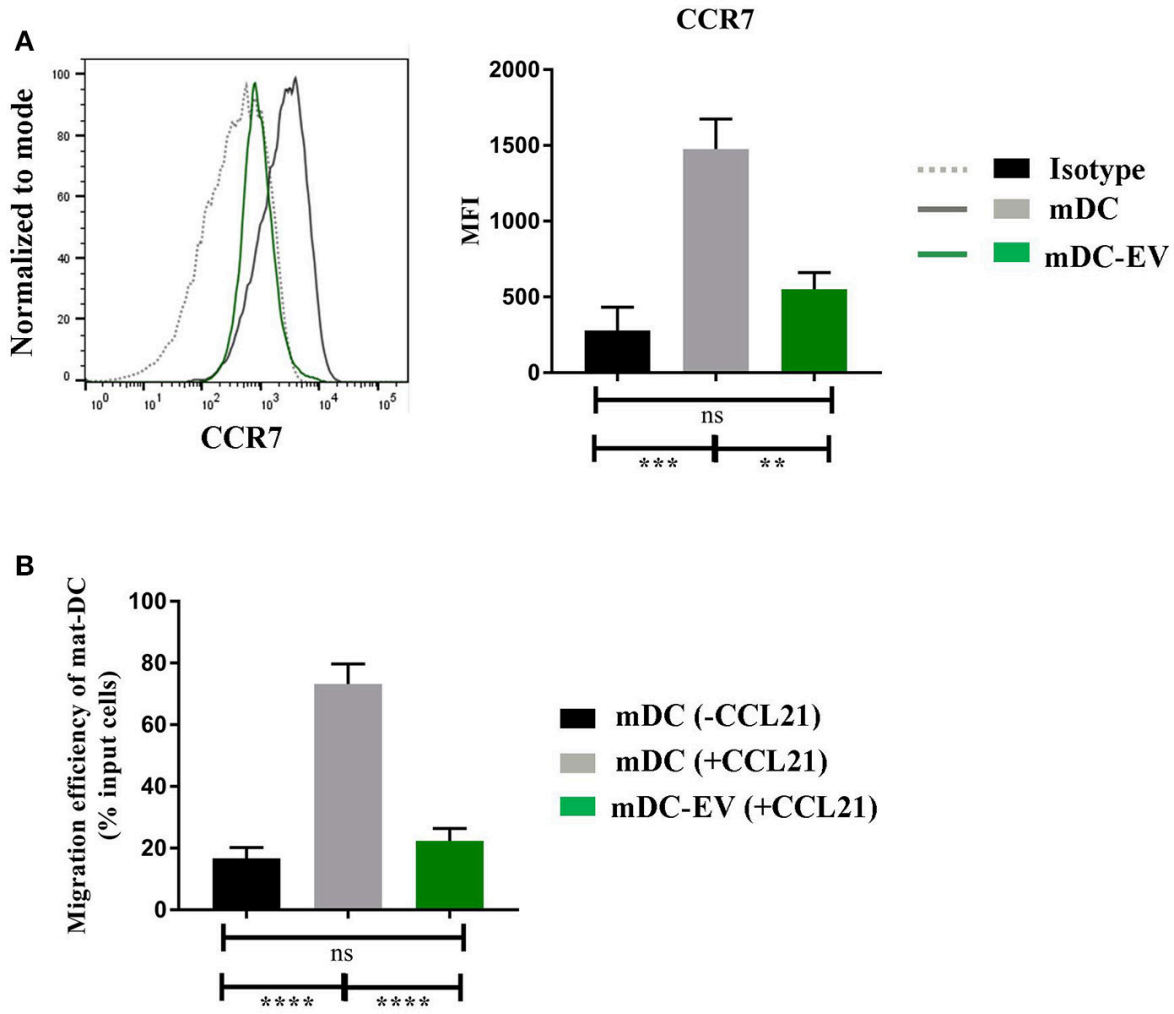
MSC-EVs impair DC migratory via suppression of CCR7 expression **(A)** Representative histograms (left) and cumulative levels (right) of CCR7 expression by mDCs and mDC-EVs. Live cells were selected prior to histograms and levels of expression were compared to isotype controls. **(B)** Migration efficiency of mDCs and mDC-EVs. Mature DCs migrated toward the medium without CCL21 served as a background control. Results were expressed as mean ± SEM of 4–6 independent experiments. **p < 0.01, ***p < 0.001 and ****p < 0.0001.

CCR7 plays an essential role in DC homing to the lymph nodes. Upon the presence of inflammatory stimuli, DCs mature and gain CCR7 expression which, by binding to its ligand CCL21 or CCL19, direct DCs to the lymph nodes for antigen presentation (31). We evaluated CCR7 expression on mDCs, with or without MSC-EV treatment, and their ability to migrate toward CCL21 using the transwell system. We detected a 3-fold lower CCR7 expression on MSC-EV treated DCs compared to the untreated counterpart (*p* = 0.003, Figure 5A). In addition, MSC-EV treated DCs showed a significantly reduced migration efficiency toward CCR7 ligand CCL21 in comparison to untreated DCs (22.4 ± 4.0% and 73.2 ± 6.4%, respectively; *p* < 0.0001, Figure 5B).

### MSC-EVs encapsulate immunomodulatory microRNAs

**Figure 6:**
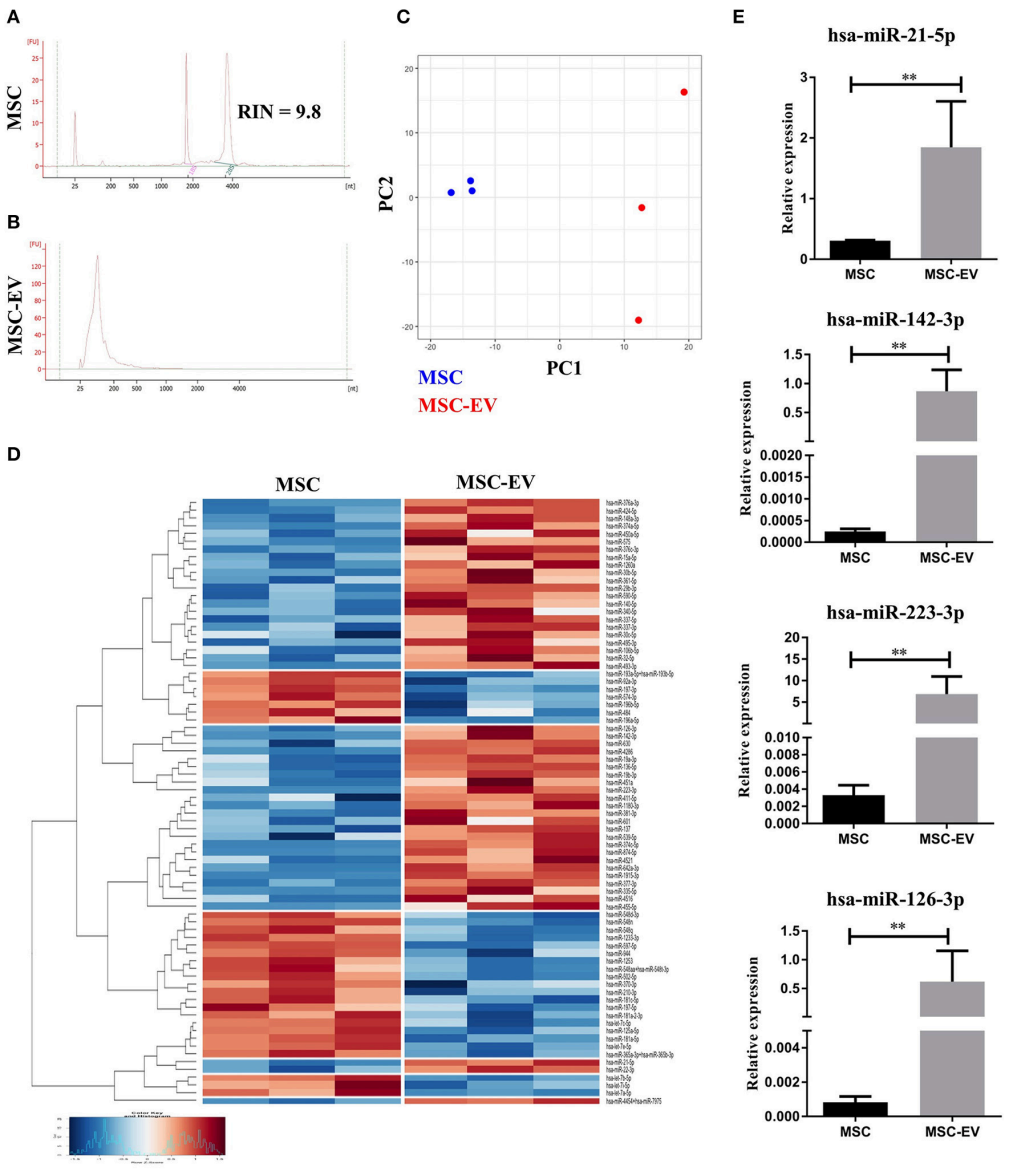
MSC-EVs encapsulate immunomodulatory microRNAs. **(A,B)** The profile of RNAs derived from MSCs and MSC-EVs as analyzed using Bioanalyzer. **(C)** Principal component analysis plot of microRNA profile of MSCs (blue) and MSC-EVs (red). **(D)** Heatmap showing the 79 differentially expressed microRNAs between MSCs and MSC-EVs. Heatmap colors represent relative microRNA expression as indicated by the color key, i.e., under-expressed (blue) and over-expressed (red). Each row is representative of one microRNA and each column one sample. RStudio™ was used to plot the results. **(E)** RT-qPCR validation of the immunomodulatory microRNAs enriched in MSC-EVs. The graphs depict relative expression levels conveyed as 2dCT and results were expressed as mean ± SEM of 3 independent experiments. **p < 0.01.

To further scrutinize the molecular mechanisms underlying MSC-EV mediated modulation of DC maturation and function, we profiled the miRNA contents in MSC-EVs in comparison to their parent cells using the NanoString technology. High quality of RNA extracted from MSCs and MSC-EVs was confirmed by bioanalyzer analysis. MSC-derived RNA showed RNA integrity numbers (RIN) >7 and exhibited single narrow peaks at the 18 and 28S ribosomal RNA (Figure 6A). MSC-EVs enclosed only small RNA species with sizes from 25 up to ~200 nucleotides. No RIN was calculated for MSC-EV derived RNA due to the absence of the ribosomal RNA peaks (Figure 6B). The microRNAs in MSCs and MSC-EVs displayed a polarized distribution as shown by the principal component analysis plot (Figure 6C). The NanoString microRNA profiling identified 79 microRNAs that were differentially expressed between MSC-EVs and MSCs. These differentially expressed miRNAs were presented in a heatmap based on a hierarchical clustering analysis (Figure 6D). Amongst these 79 miRNAs, 49 were enriched in MSC-EVs while the remaining 30 were enriched in the parent cells. MiRNAs with known effect on DC maturation and function, including miR-21-5p, miR-142-3p, miR-223-3p, and miR-126-3p, were detected within the top 10 enriched miRNAs in MSC-EVs (Table 1). These microRNAs were validated using RT-qPCR in an independent cohort of samples and showed significantly higher expression levels in MSC-EVs compared to parent cells (Figure 6E), suggesting a potential selective packaging of these microRNAs into EVs, which may serve as a vehicle to deliver the miRNA contents to the target cells. A detailed list of the 79 differentially expressed miRNAs detected in MSC-EVs and respective parent cells are provided in Supplementary Table S1.

**Table 1:** Top 10 microRNAs in MSC-EVs.

**microRNA**	**Counts**	**SEM**
hsa-miR-4454 + hsa-miR-7975	173, 824.7	501.6
hsa-miR-4286	8, 182.4	2.3
hsa-miR-21-5p	6, 957.7	122.5
hsa-miR-223-3p	2, 029.6	0.0
hsa-miR-142-3p	1, 659.4	0.2
hsa-miR-22-3p	1, 631.4	72.3
hsa-miR-29b-3p	1, 378.2	34.6
hsa-miR-630	1, 232.5	4.7
hsa-miR-126-3p	1, 156.7	5.2
has-miR-1260a	675.9	144.4

*Top 10 microRNAs are listed according to their expression levels (count number) in MSC-EVs as detected by the Nanostring miRNA profile (n = 3). Counts represent the geometric mean of copy numbers detected in each analyzed sample ± SEM*.

### Transfection of DCs with miRNA-21-5p partially mimics the function of MSC-EV treated DCs

**Figure 7:**
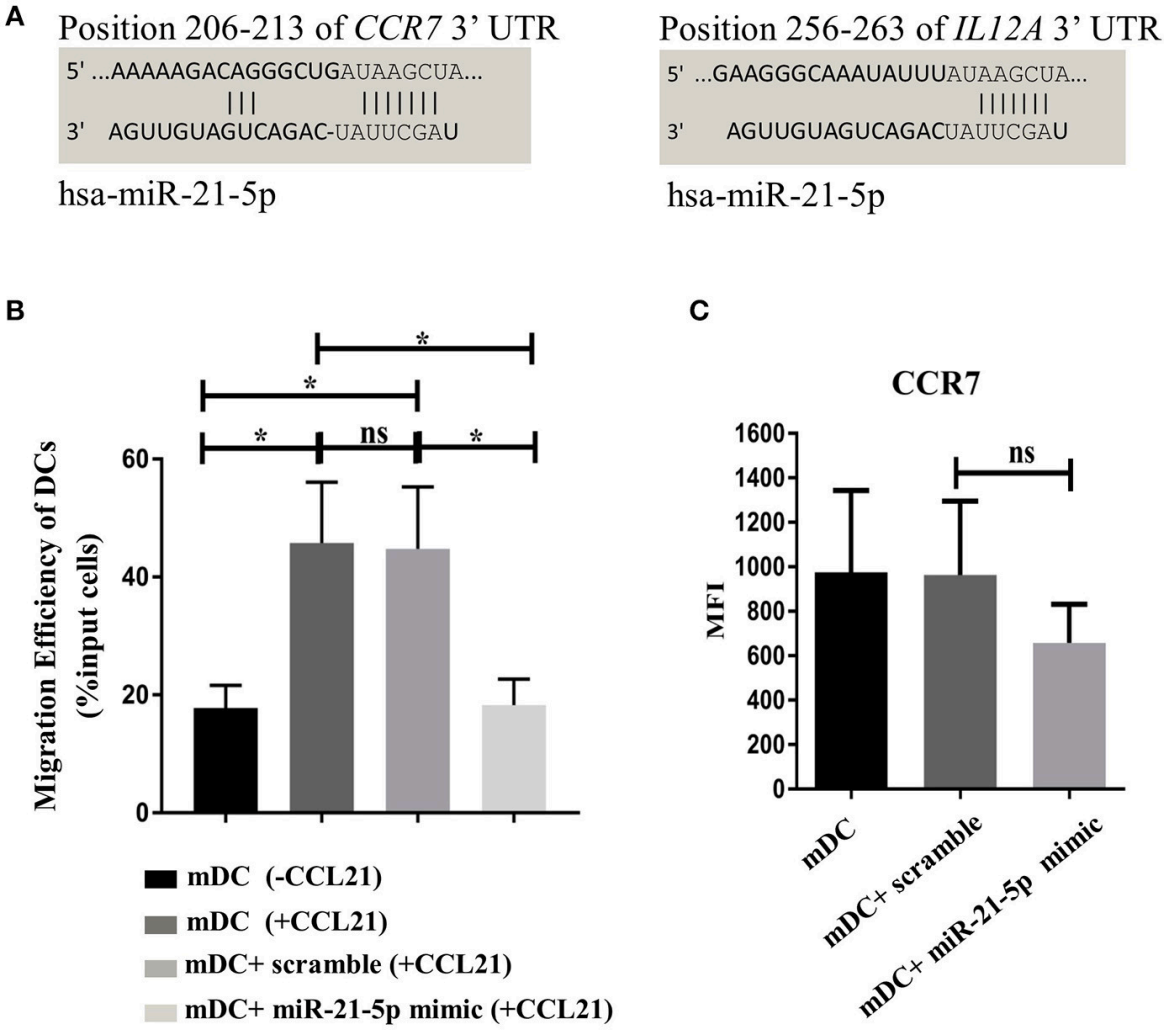
Transfection of DCs with miR-21-5p partially mimics the function of MSC-EV treated DCs. **(A) ***In silico* analysis of miR-21-5p targets revealed that this microRNA directly targets *CCR7* and *IL12A* genes. Targets for miR-21-5p were queried using miRSystems and graphic representations and complementarity information of miR-21-5p with CCR7 and IL12A genes were obtained from the online database http://www.targetscan.org/vert_72/. **(B)** Migratory capacity of non-transfected DCs and DCs transfected with MiR-21-5p mimics or scramble controls, as detected using a transwell system. DCs migrated toward medium without the chemoattractant served as a background control. **(C)** CCR7 expression was analyzed by flow cytometry. Live cells were selected prior to histograms and levels of expression were compared to the isotype control. Results were expressed as mean ± SEM of 4-6 independent experiments. *p < 0.05.

MicroRNA-21-5p is one of the most enriched miRNAs enclosed in MSC-EVs (Table 1). Target analysis revealed that the *CCR7* gene can be targeted by miR-21-5p (Figure 7A), suggesting the enriched miRNA-21-5p in MSC-EV may function as a potential molecular mechanism underpinning MSC-EV mediated impairment of DC migration and function. To confirm this hypothesis, we examined whether overexpression of miR-21-5p in DCs could recapitulate the effect of MSC-EV treatment by transfecting DCs with a miR-21-5p mimic and a fluorescently labeled miRNA scramble on day 3 of DC generation culture using a lipid based transfection method as previously reported (28). Post-transfection assessment showed the highest transfection efficiency at 72 h with comparable cell viability between transfected and non-transfected cells (Supplementary Figures S4A–C). Overexpression of miR-21-5p was confirmed by RT-qPCR compared to the non-transfected and scramble miRNA controls (*p* < 0.05; Supplementary Figure S4D). Functionally, miR-21-5p mimic transfected DCs exhibited a significant decrease in their migratory capacity toward CCL21 in comparison to non-transfected and scramble miRNA transfected controls, with a migration efficiency of 18.25 ± 4.43%, 44.75 ± 10.33%, and 44.75 ± 10.76%, respectively (*p* < 0.05 for both; Figure 7B). MiR-21-5p mimic transfected DCs also showed a trend toward a reduction in CCR7 expression compared to the controls, although a statistical significance was not achieved (Figure 7C). In addition, a tendency of secreting lower levels of IL-6 and IL-12p70 was observed in MiR-21-5p mimic transfected DCs compared to both controls, although a statistical significance was not reached (*p* > 0.05 for all; Supplementary Figure S4E).

## Discussion

MSC-EVs have been reported to recapitulate the intrinsic regenerative and immunomodulatory properties of MSCs (32–35). So far, little knowledge is available about the influence of MSC-EVs on DC modulation and associated potential mechanistic pathway by which MSC-EVs exert their modulatory effects on DCs.

We have demonstrated for the first time that MSC-EVs are able to impede DC maturation and modify DC function, in a similar manner to that previously reported with MSCs (21, 36, 37). Exposure to MSC-EVs has attenuated antigen uptake by immature DCs and rendered mature DCs with a semi-mature phenotype after LPS stimulation, characterized by intermediate level expression of CD83, CD38, and CD80 that was higher than immature DCs but lower than untreated mature DCs. This phenotype change was accompanied with a functional shift in DC cytokine production profile from inflammatory to immunoregulatory. MSC-EVs have been reported to induce regulatory macrophage phenotype change and skew their cytokine production toward a regulatory profile with increased production of anti-inflammatory cytokines and downregulation of inflammatory cytokine production (11, 33, 38). A recent study by Tung et al. (39) also demonstrated that regulatory T cell-derived EVs (Treg-EVs) induced anti-inflammatory cytokine secretion by DCs, with an increased IL-10 and decreased IL-6 production following LPS stimulation (39). It is well-recognized that regulatory T cells (Tregs) and MSCs share similar immunomodulatory properties. The research findings from Treg-EVs tend to corroborate our observations from MSC-EVs. The discrepancy in the ability of Treg-EVs and MSC-EVs to influence the expression of the co-stimulatory molecule CD80 and the difference in Treg-EV and MSC-EV induced IL-10 production by DCs endorse the notion that although Tregs and MSCs share similar immunomodulatory properties they may exert their modulatory effects on DCs through different mechanistic pathways.

It is clear that DCs orchestrate the immune system through both cell-cell contact and cytokine secretion pathways. IL-12 and TGF-β play critical roles in shaping immune responses during antigen presentation and influencing cell-fate decisions relating to naïve T cell differentiation into the appropriate T cell subtypes in response to the environment challenge. DCs are one of the key producers of IL-12, which dominantly target T cells by inducing T cell IFNγ production mainly through STAT4 (40). Our observation of a decreased IL-12-p70 production by MSC-EV treated DCs suggests that MSC-EV treated DCs may skew the balance of Th1 and Th2 effector T cells, in favor of the latter, as evidenced in our further finding that T cells stimulated with MSC-EV treated DCs had a markedly reduced secretion of IFNγ. It is important to confirm the T cell subsets derived from the naïve T cells stimulated with MSC-EV treated DCs in future studies, including demonstrating IL-4 and IL-5 production by T cells stimulated by MSC-EV treated DCs. TGF-β is an important cytokine playing fundamental roles in the regulation of immune responses during homeostasis and disease. In addition to the direct effect on Th1 and Th2 effector responses, TGF-β promotes immunosuppression via direct induction of Foxp3 expressing Tregs both in the thymus and the periphery (41, 42). The potency of MSC-EVs in inducing Treg production was previously demonstrated in mouse models of GvHD and type 1 diabetic patients (43–45). MSC-EVs ameliorated GvHD symptoms and increased survival in a murine model of GvHD via the induction of CD4^+^CD25^+^FoxP3^+^ T cells in an APC-mediated pathway (43). The therapeutic use of MSC-EVs for the treatment of therapy-refractory GvHD in humans has also been observed showing that MSC-EV treatment was able to ameliorate GvHD symptoms with evident inhibition of inflammatory cytokine secretion (35).

Controversial findings have been reported with regard to the ability of MSC-EVs to suppress T cell proliferation. MSC-EVs have induced a significant suppression of the proliferation of CD3^+^ T cells stimulated directly with anti-CD3/CD28 in the absence of DCs (8). Co-cultures of autologous T cells with MSC-EV conditioned DCs pulsed with a specific auto-antigen showed no significant reduction in T cell activation and proliferation when re-challenged with the same antigen (46). In our study comparable levels of allogeneic T cell proliferation were observed following the stimulation with either untreated or MSC-EV treated DCs *in vitro*, suggesting that MSC-EVs may influence T cell response predominantly via skewing T cell cytokine production profile as evidenced in our observation of a decreased IFNγ and IL-6 secretion by the T cells stimulated with MSC-EV treated DCs. It is worth noting that, despite the inability of MSC-EV treated DCs to suppress T cell proliferation in the *in vitro* experiment, our finding that MSC-EV treated DCs had decreased CCR7 expression and reduced ability to migrate toward the CCR7 ligand CCL21 indicates the possibility that MSC-EV treated DCs may be able to dampen down inflammatory T cell responses *in vivo* due to impaired migration to secondary lymphoid tissues. This notion is supported by previous observations from murine *in vivo* models showing that intravenous administration of MSCs decreased the number of CCR7 expressing DCs in the draining lymph nodes and hindered local antigen priming of CD4^+^ T cells (22) Interestingly, in our study when MSC-EVs were added directly to the co-culture of DCs and allogeneic T cells a diminished T cell proliferation was detected (Supplementary Figure S1), suggesting that MSC-EVs may also exert direct effect on T cells under certain conditions, as shown in other studies (8, 10). Although the mechanisms of MSC-EV mediated suppression of T cell proliferation are not fully understood, published data suggests that these vesicles induce T cell apoptosis (11).

One of the most striking phenotype changes in MSC-EV treated DCs is the reduction in CD38 expression. CD38 is an ectoenzyme that catalyzes the synthesis of cyclic adenosine diphosphate ribose, which initiates transmembrane signaling upon engagement with CD31 (47). It induces the expression of CD83 and IL-12 production in DCs via NF-κB pathway (48, 49). CD38 is also laterally associated with CCR7, therefore an important regulator of DC migration (48). In addition, data from a murine model showed that CD38^−/−^ immature DCs are less efficient in taking up antigens (50). Taken together, MSC-EV mediated suppression of CD38 expression in DCs may be an initiating upstream event contributing directly to the reduced phagocytic capacity, decreased CD83 expression and IL-12 production, as well as impaired CCR7 expression and CCL21 driven migration, as observed in this study. Future studies into the relevant molecular signaling pathways may provide further insight to uncover the complex interactive signaling networks involved in MSC-EV mediated DC modulation.

Another key novel finding from this study is that microRNAs enclosed in the MSC-EVs may represent a potential mechanism by which MSC exert their modulatory role in DCs. Increasing evidence suggests that MSC-EV enclosed miRNAs are important effectors for the biological and therapeutic effect of these vesicles (6, 34, 51, 52). Our study identified four highly enriched microRNAs with essential roles in DC development and function in MSC-EVs, including miR-21-5p, miR-142-3p, miR-223-3p, and miR-126-3p. Based on the miRNA profile of MSC-EVs and the effect of these vesicles on DC maturation and function, we performed target analysis using the miRSystem online software which retrieves both validated and predicted microRNA targets from multiple online databases (53). The analysis revealed that miR-223-3p targets the *CD83* gene, while miR-142-3p has been experimentally observed as an inhibitor of IL-6 expression (54). Recent evidence also showed that Treg-EV enclosed miR-142-3p can be acquired by DCs resulting in the induction of a tolerogenic phenotype in DC (38). Moreover, miR-126-3p targets genes upstream TLR signaling, such as *Tsc1*, a negative regulator of the mTOR kinase (55) and miR-29b targets Bcl-2 gene, which is involved in the maintenance of DC longevity *in vivo* (56). MiR-21-5p is one of the most highly expressed microRNAs in MSC-EVs. It has been experimentally observed to directly target *IL-12A*, inhibit IL-12p35 production, as well as indirectly drive *IL-6* degradation, and promote the expression of TGF-β1 and IL-10 (57–59). MiR-21-5p has also been experimentally observed to directly target *CCR7* gene for degradation. In DCs miR-21 was shown to have a highly conserved target region in *CCR7* 3′UTR and to be significantly down-regulated upon DC maturation (60). Lentiviral transfection of DCs with miR-21 has demonstrated a reduction of *CCR7* expression after LPS maturation. In our study, transfection of DCs with miR-21-5p mimics has significantly diminished DC migration toward the CCR7 ligand CCL21, accompanied with a tendency toward a decreased CCR7 protein expression and reduced proinflammatory cytokine production. Given the high concentration of miR-21-5p detected in MSC-EVs and the critical roles it plays in DC function, it is plausible to hypothesize that delivery of miR-21-5p via secreted EVs may be an important potential mechanistic pathway underpinning MSC mediated modulatory effects on DCs. However, further studies would be needed to demonstrate the direct delivery of MSC-EV enclosed miR-21-5p to DCs and the impact of other relevant microRNAs, as well as the bioactive factors beyond microRNAs, should be duly recognized. Furthermore, MSCs secrete a heterogeneous population of vesicles. It is possible that distinct vesicles purified from MSC conditioned media exhibit distinct immunomodulatory properties (61, 62). However, assessing the functionality of EV subpopulations may not be succeeded until better and more high throughput purification methodology is developed for EV purification.

In summary, our findings suggest that MSC-EVs are able to recapitulate MSC mediated DC modulation and MSC-EV enclosed microRNAs may represent a potential novel mechanism through which MSCs modulate DC functions. As MSCs are currently used in clinical trials to treat numerous immune dysregulation associated diseases, our data provide novel evidence to inform potential future application of MSC-EVs as a cell-free therapeutic agent.

## Ethics statement

All human samples used in this study were collected from healthy volunteers. This study was carried out in accordance with the recommendations of Newcastle and North Tyneside Research Ethics Committee (REC 14/NE/1136) with written informed consent from all subjects. All subjects gave written informed consent in accordance with the Declaration of Helsinki. The protocol was approved by the Newcastle and North Tyneside Research Ethics Committee (REC 14/NE/1136). All procedures were performed in accordance with the relevant guidelines and regulations.

## Author contributions

MR and XW conceived the research idea, designed the experiments, and wrote the manuscript. MR, EM, and LN performed the experiments and analyzed the data. KG assisted with the NanoString microRNA profiling. AD contributed to the initial research planning and provided valuable guidance throughout the project.

### Conflict of interest statement

The authors declare that the research was conducted in the absence of any commercial or financial relationships that could be construed as a potential conflict of interest.
