# Supplementary material for: Mesenchymal Stromal Cell-Derived Extracellular Vesicles Attenuate Dendritic Cell Maturation and Function
Source: Front Immunol. 2018 Nov 9;9:2538. doi: 10.3389/fimmu.2018.02538 (PMC6237916; doi:10.3389/fimmu.2018.02538)
Supplement: Supplementary file 2 [file Data_Sheet_1.docx]

***Supplementary Material***

**Mesenchymal stromal cell-derived extracellular vesicles attenuate dendritic cell maturation and function**

Monica Reis, Emily Mavin, Lindsay Nicholson, Kile Green, Anne M. Dickinson and Xiao-nong Wang*

*Correspondence: Xiao-nong Wang: [x.n.wang@newcastle.ac.uk](mailto:x.n.wang@newcastle.ac.uk)

**Supplementary Data**

**Supplementary Materials and Methods**

**Characterization of bone marrow derived MSCs**

MSCs were characterized as recommended by the ISCT criteria^22^. At passage 3, MSCs were assessed for their plastic adherent feature and spindle-shaped morphology, expression of a defined panel of cell surface markers and tri-lineage differentiation ability. For phenotype analysis by flow cytometry, MSCs (3x10^5^ cells) were stained with CD14-, CD19-, CD34- and CD45- FITC, along with CD73- PE, CD105-APC, CD90-PerCPCy5.5 and HLA-DR-APC. Appropriate isotype controls were used as negative controls. DAPI was used to exclude the dead cells. Differentiation into adipo-, osteo- and chondrocytes was performed *in vitro*. For adipogenesis, MSCs were seeded at a density of 1x10^5^ cells/ml in a 6-well plate. When ∼100% confluent, StemMACS^TM^ adipogenic medium (Miltenyi) was added to the wells. Cells were maintained in culture for 21 days with medium replacement every 3 days. The differentiation potential for adipogenesis was assessed by 0.3% Oil-Red-O staining after fixation in 10% Neutral buffered formalin. Osteogenesis was carried out by seeding 3 x 10^4^ cells/well in StemMACS^TM^ OsteoDiff medium (Miltenyi).  Cells were maintained in medium for 21 days with media replaced twice weekly. The differentiation potential for osteogenesis was assessed by the alkaline phosphatase and von Kossa staining after fixation in 10% Neutral buffered formalin. Chondogenesis was performed by re-suspending 2x10^5^ cells in pre-warmed StemMACSTM ChondroDiff medium (Miltenyi). Cells were centrifuged and incubated as a pellet for 24 days with medium replacemed twice weekly. Production of glycosaminoglycans was measured by staining with 0.1% Alcian blue (Sigma-Aldrich). After staining, differentiated cells were visualized under a Zeiss light microscope.

**Optimization of MSC-EV dose in T cell proliferation assay**

The optimized concentration of MSC-EVs was determined by adding a titrated dose of MSC-EVs to a mixed lymphocyte reaction consisting of allogeneic CD3^+^ T cells primed with mDCs. CD3^+^ T cells alone were used as negative controls. After a 5 day MLR, T cell proliferation was assessed using thymidine incorporation assay, following all local rules for safe use of radioactive material, where cells were pulsed with 10 µl of 3H-Thymidine (0.185 MBq/ml, Perkin Elmer) and incubated for 16h. The cells were then harvested onto a filter mat with an INOTECH cell harvester (CH-5610 Wholen) and the levels of proliferation was assessed by counting radioactivity using a Direct Beta Counter (MATRIX 9600, Packard Instrument).

**Optimization of DC migration**

To optimize the migration conditions of DCs using the transwell system, mature DCs at a density of 1x10^5^ cells/insert (input cells) were seeded on an 8 µm pore insert and medium supplemented with either CCL21 or CCL19 (250 ng/ml, R&D systems) were added to the lower chamber. DC migration was assessed by harvesting cells from the bottom chamber at 2, 6 and 24h of incubation at 37^o^C in a 5% CO_2_ humidified atmosphere. The migrated cells were manually counting using a Neubauer chamber. Migration efficiency was expressed as the percentage of input cells. Mature DCs migrating towards medium without chemokines CCL19 and CCL21 was used as negative control.

**Supplementary Figures**

**Supplementary Figure S1: Optimization of MSC-EV dose in T cell proliferation assay.** CD3^+^ T cells were co-cultured with allogeneic-DCs in the presence or absence of a titrated dose of MSC-EVs (2, 4 and 20 μg/ml). Co-culture was maintained for 5 days after which T cell proliferation was assessed by 3H-thimidine incorporation. MSC-EVs inhibited T cell proliferation in a dose dependent manner, with the highest inhibition detected when 20 μg/ml of MSC-EVs was used. The MSC-EV concentration of 20 μg/ml was used for all functional analysis throughout this study.

**
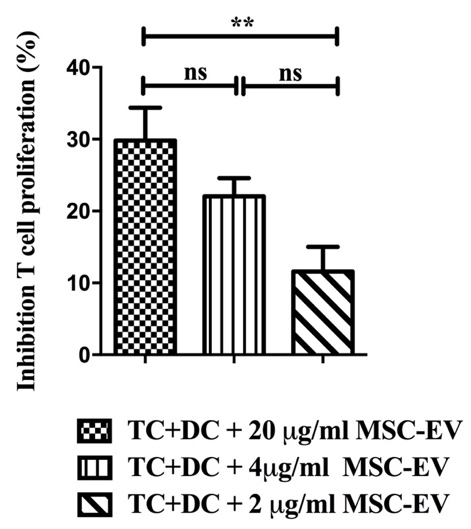
**

**Supplementary Figure S2: Optimization of DC migration conditions.** Mature DCs were seeded at a density of 1.5 x 10^5^ cells on 8μm pore upper chamber. Medium supplemented with either CCL21 or CCL19 was added to the lower chamber and DC migration towards CCL21 and CCL19 was assessed at 2h, 6h and 24h. Medium without chemokine in the lower chamber was used as a background control. The highest migration efficiency was detected when DCs migrated towards CCL21 supplemented media after 24h of incubation in the transwell system. This condition was used to examine DC migratory abilities throughout this study

**
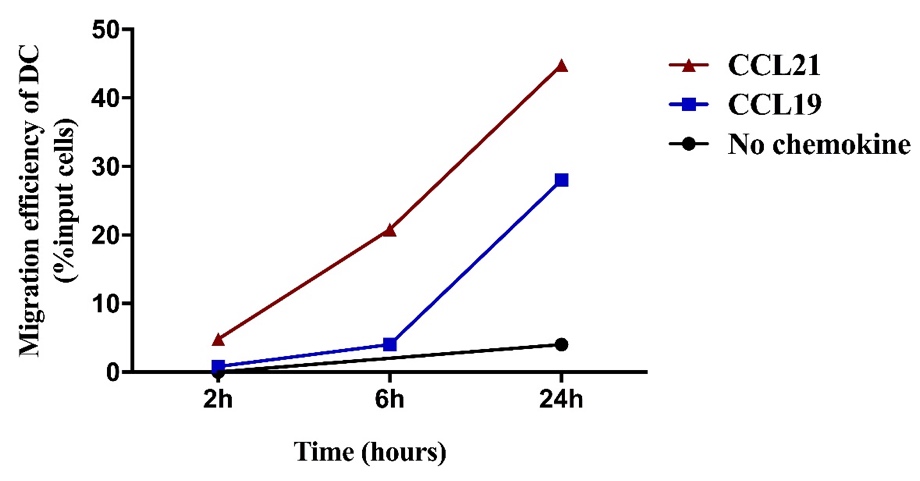
**

**
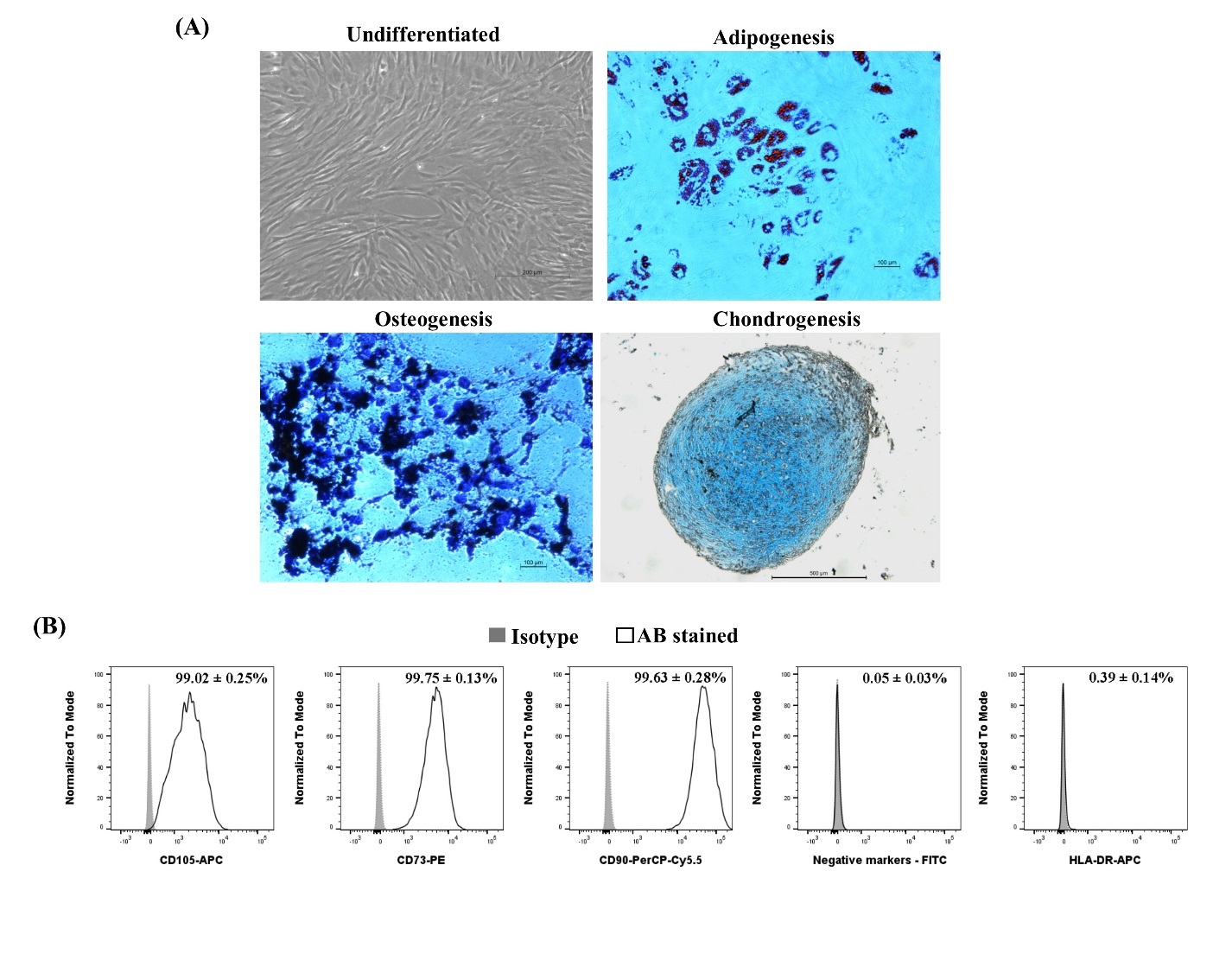
Supplementary Figure S3: Characteristics of MSCs.** MSCs used in this study were confirmed being in compliance with the requirements of the International Society for Cellular Therapies. **A)** Representative images showing the characteristic spindle shaped morphology and tri-lineage differentiation capacity of MSCs at passage 3. **B)** Representative histograms of passage 3 MSCs showing greater than 95% of cells expressed CD73, CD90 and CD105, and less than 2% of cells expressed lineage specific markers CD14, CD19, CD34 and CD45 and HLA-DR.

**
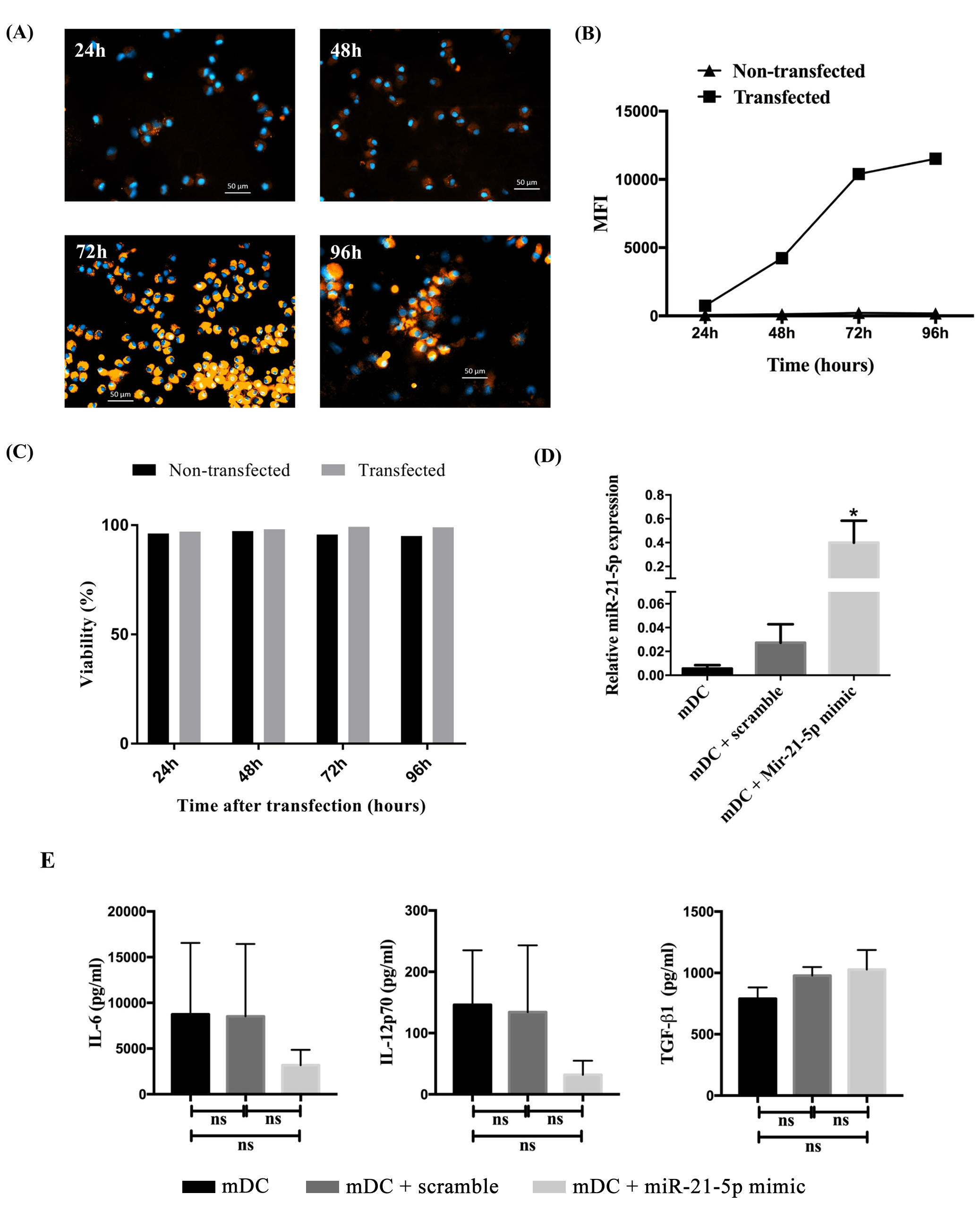
**

**Supplementary Figure S4: The efficiency of DC transfection.** On day 3 of the DC generation culture DCs were transfected with the Dy547 conjugated miRIDIAN microRNA has-miR-21-5p mimic, scramble (siRNA control) or the transfection reagent (non-transfected) using a lipid-based transfection method. Transfection efficiency was assessed at 24, 48, 72 and 96h after transfection. **A)** Fluorescence intensity observed using microscopy. **B)** The mean fluorescence intensity of Dy547 at 24, 48, 72 and 96h detected by flow cytometry. **C)** Cell viability compared between the transfected and non-transfected DCs. **D)** The efficiency of DC transfection was confirmed by real time qPCR. **E)** Preliminary data showing the cytokine production by miR-21-5p transfected DCs. Results were expressed as mean±SEM of 3 independent experiments. *p<0.05.
